# OPPORTUNITIES AND CONSIDERATIONS WHEN WORKING WITH AGING PET OWNERS: THE DEVELOPMENT OF GUIDES FOR PROFESSIONALS

**DOI:** 10.1093/geroni/igad104.2072

**Published:** 2023-12-21

**Authors:** Jessica Bibbo, Sarah Nicolay, Justin Johnson

**Affiliations:** Benjamin Rose Institute on Aging, Cleveland, Ohio, United States; Benjamin Rose Institute on Aging, Cleveland, Ohio, United States; Benjamin Rose Institute on Aging, Cleveland, Ohio, United States

## Abstract

Pets can be an important relationship in the lives of older adults and are often a factor in older adults’ behaviors and decisions. Guided by the stress process model, the aim of this study was to understand the benefits, challenges, and resources of pet ownership geriatric professionals encountered in their work. An interdisciplinary sample (N=462, 89.13% female, Mage=53.02, SDage=12.18) of geriatric professionals (i.e., people working with older adults [OA], persons with dementia [PWD], and/or caregivers [CG]) working in healthcare, social services, and private and community-based services (e.g., long-term care, housing) completed an online survey on pet ownership issues they had encountered in their work. A convergent parallel approach was used in data analysis. Quantitative data were analyzed with descriptive statistics, t-tests, and repeated measures ANOVAs. Qualitative data were analyzed using a directed content analysis to create codes and themes. The benefits of pet ownership were wellbeing (e.g., positive mental and physical health) and connections. Pet care was the most common challenge (e.g., basic care, financial), caregiver strain was unique to CGs. Other challenges were planning, safety (e.g., falls, pet behavior), and the pet being a barrier to care. The companionship directly experienced by OA and PWD was the central resource of pet ownership. The results were synthesized to develop guides for professionals on the benefits, challenges, opportunities, and considerations when working with older adult pet owners (including PWD) and another was created for professionals working with caregivers aging pet owners (living with and without dementia).

